# Administration of signalling molecules dictates stem cell homing for *in situ* regeneration

**DOI:** 10.1111/jcmm.13286

**Published:** 2017-08-02

**Authors:** Xuan Li, Xiao‐Tao He, Yuan Yin, Rui‐Xin Wu, Bei‐Min Tian, Fa‐Ming Chen

**Affiliations:** ^1^ State Key Laboratory of Military Stomatology and National Clinical Research Center for Oral Diseases Department of Periodontology School of Stomatology Fourth Military Medical University Xi'an China

**Keywords:** stem cell homing, chemokines, controlled release, *in situ* tissue engineering, cell modification

## Abstract

*Ex vivo‐*expanded stem cells have long been a cornerstone of biotherapeutics and have attracted increasing attention for treating intractable diseases and improving tissue regeneration. However, using exogenous cellular materials to develop restorative treatments for large numbers of patients has become a major concern for both economic and safety reasons. Advances in cell biological research over the past two decades have expanded the potential for using endogenous stem cells during wound healing processes, and in particular, recent insight into stem cell movement and homing has prompted regenerative research and therapy based on recruiting endogenous cells. Inspired by the natural healing process, artificial administration of specific chemokines as signals systemically or at the injury site, typically using biomaterials as vehicles, is a state‐of‐the‐art strategy that potentiates stem cell homing and recreates an anti‐inflammatory and immunomodulatory microenvironment to enhance *in situ* tissue regeneration. However, pharmacologically coaxing endogenous stem cells to act as therapeutics in the field of biomedicine remains in the early stages; its efficacy is limited by the lack of innovative methodologies for chemokine presentation and release. This review describes how to direct the homing of endogenous stem cells *via* the administration of specific signals, with a particular emphasis on targeted signalling molecules that regulate this homing process, to enhance *in situ* tissue regeneration. We also provide an outlook on and critical considerations for future investigations to enhance stem cell recruitment and harness the reparative potential of these recruited cells as a clinically relevant cell therapy.

## Introduction

Our ageing population is facing an increasing burden of many age‐related degenerative and ischaemic diseases that currently are primarily treated with drugs designed to mitigate symptoms or with resection and reconstructive surgery in certain clinical scenarios 1, 2. Notably, the transplantation of tissues of either autogenic or allogenic origin has provided cures for various types of tissue deficiency, dysfunction and damage 3. Meanwhile, organ allotransplantation is routine and successful in clinical practice; this technique has saved the lives of numerous patients suffering from organ failure and improved the quality of life of many more (*e.g*. see 4, 5, 6, 7, 8, 9). Unfortunately, due to immunologic barriers and limited donor availability, such therapeutic strategies are applicable to only a small range of clinical scenarios or a small fraction of patients 10. Together with the ever‐increasing demand for organ transplants, the gap between supply and need continues to widen 11. For many conditions, even if tissue/organ allotransplantation is technically feasible and performed during the correct stage, morbidity and mortality due to treatment‐associated complications (*e.g*. graft‐versus‐host disease) remain unacceptably high, and the overall success rates are frustratingly low 12, 13. Therefore, innovative applications of novel cell therapies and tissue engineering to regenerate damaged tissue structures and to restore lost tissue function represent major frontiers for modern biomedicine, although the most satisfactory and successful stem cell therapy strategy remains to be explored and optimized 1, 14, 15, 16.

During the last decade, accumulating knowledge regarding endogenous mechanisms for the self‐repair of injured tissue has paved the way for the design of *in situ* regenerative approaches to achieve complete tissue repair 17, 18, 19. Although living tissues can possess inherent mechanisms that instruct stem cells to home to damaged areas to promote self‐repair, such staggering endogenous processes unfortunately cannot provide a universal regenerative solution 20. One key to potentiating and accelerating the body's own repair capacity is the proficient homing of endogenous stem cells into injury sites *via* the prolonged and controlled delivery of signalling molecules during the initial stage of wound healing 19, 21, 22. In this context, chemokines powerfully influence cell mobilization and homing, and artificially amplifying the doses or concentrations of particular chemokines at the site of damage represents an efficient approach to actively increasing the homing of host stem cells, thus augmenting *in situ* tissue regeneration 17, 19, 23, 24. The stem or progenitor cells in the local niche neighbouring the tissue defect are normally too few in quantity to strongly affect the intrinsic repair processes; therefore, in most cases of *in situ* tissue regeneration, it is advisable to actively mobilize mesenchymal stem cells (MSCs) from a central cell niche, such as the bone marrow (BMMSCs), into the peripheral blood system and to target these cells for therapeutic strategies by replenishing the local cell niche and/or for direct participation in regeneration 17, 18, 19, 21, 22, 23, 24, 25.

Similar to strategies applied to improve the homing and engraftment of exogenously transplanted cellular materials in recipient tissues (*e.g*. see 26, 27, 28), increasing the ability of the damaged site to recruit host cells and the extent to which the damaged site allows the recruited cells to exert their function are also critically important for ensuring the outcome of any endogenous regenerative procedures 29. Both goals can commonly be achieved by the implantation of a well‐devised material platform 18. The biological evidence underlying *in vivo* cell movement and its related mechanisms of action in self‐repair have been reviewed elsewhere; the readers are pointed to several previously published reviews for more information 18, 19, 21, 22, 23, 24, 25. In this context, protein delivery plays a critical role in the presentation and release of signalling molecules that target cell mobilization, homing and engraftment, together leading to tissue regeneration 30, 31. In this review, we briefly outline the identified and suggested signalling molecules that can affect the efficacy of cell migration, with a particular emphasis on how they are administered to direct stem cell homing and enhance the *in situ* regeneration process. We also critically evaluate their roles in biomaterials‐based stem cell homing and accommodation.

## Steering endogenous cell populations for therapeutics

**Figure 1:**
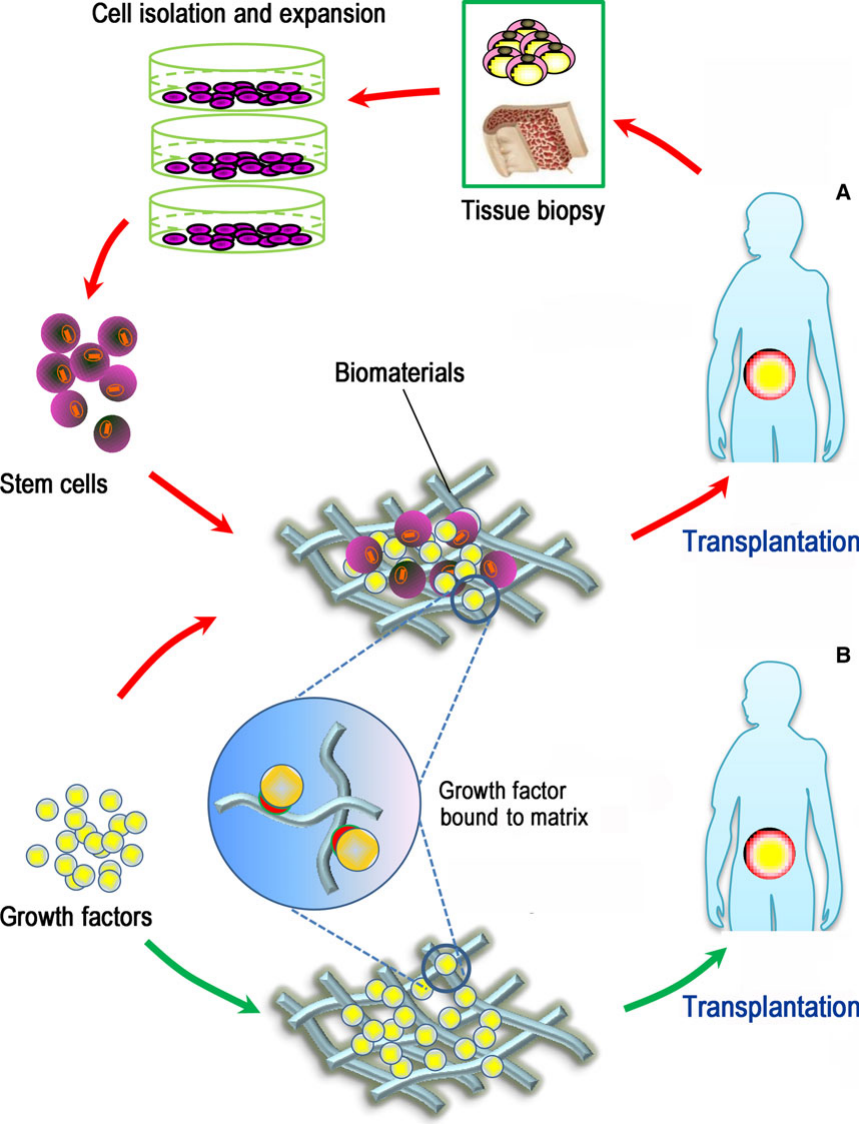
Schematic representation of cell‐based and cell‐free regenerative approaches. (**A**) A cell‐based approach (red arrows) involves harvesting stem cells from the tissue biopsy and expanding them *in vitro*; those cells, alone or in combination with biomaterials and selected signalling biomolecules, are then transplanted into the patient to regenerate damaged/diseased tissue. (**B**) In contrast, a cell‐free approach (green arrows) is used to harness endogenous stem cells for therapeutic regeneration using biomaterials bound with growth factors and thus does not require *ex vivo* cell manipulation and *in vivo* cell transplantation.

**Figure 2:**
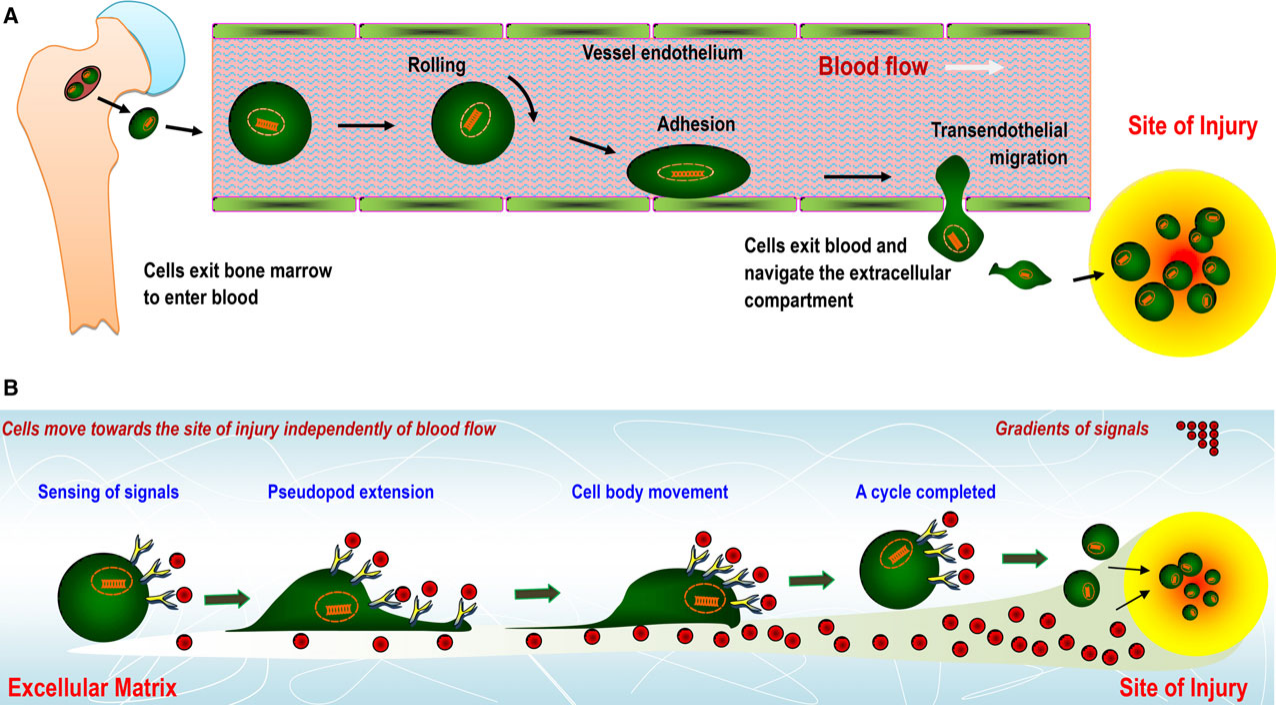
Schematic representation of stem cell movement and homing in the body in response to gradients of guidance cues (*e.g*. growth factors and/or chemokines) that are administered artificially or released by the tissue in response to injury or inflammation. (**A**) Cells are mobilized from the bone marrow and enter the blood. With the aid of blood flow, they traffic towards a distant target site and finally exit the microvascular systems *via* a multistep adhesion cascade. (**B**) Cells navigate extravascularly across the extracellular compartment to reach an injured site and participate in tissue regeneration.

Given the roles of pluripotent and tissue‐restricted stem cells in maintaining and replenishing tissues, the potential activation of these cell populations for the development of novel therapies has fuelled a veritable explosion of studies in the emerging arena of biological therapeutics and regenerative medicine 16, 32, 33. The basic strategy of stem cell‐based regeneration is based on a combination of autologous or allogeneic stem cells with a matrix template incorporating suitable growth factors, thus yielding cell/tissue constructs that can be utilized for reparative procedures in patients 1 (Fig. 1A). However, in addition to the expensive and time‐consuming *in vitro* cell expansion procedures, several other technical hurdles must be addressed before the clinical utility of such stem cell therapies for combating human diseases can be realized 15, 34. As an alternative to cell transplantation, tissue regeneration can also be achieved using a cell‐free approach that obviates the need for delivering stem cells from an exogenous source, thereby qualifying this technique for broader applications (Fig. 1B) (*e.g*. see 35, 36, 37, 38). Increasing evidence indicates that the use of bioactive molecules and material matrices may harness the therapeutic potential of endogenous stem cells and, hence, unlock the innate power of the body to promote *in situ* tissue regeneration 17, 18, 19, 39, 40. To therapeutically target‐specific niche features (*e.g*. cell‐matrix and cell‐cell contacts), several cell‐free tissue‐engineering approaches have been developed based on biomaterials and the local or systemic administration of biologics or small molecules that may reverse the impaired regenerative microenvironment due to disease and/or induce homing of circulating stem cells for regeneration 40, 41. In these cases, endogenous cells residing in the central niches (*e.g*. the bone marrow) are activated in the circulation by extracellular signals and reach the injury site with the aid of blood flow. These stem cells can be arrested within the local vasculature in response to injury and transmigrate across the endothelium to replenish the local niche and/or participate in regeneration (Fig. 2A). In contrast, quiescent stem cells within local niches neighbouring the injury site can also be recruited to exert their reparative effects for the regeneration of new tissue. In this context, cells can reach the site of injury independently of blood flow (Fig. 2B) 19. During any regenerative event, the two types of directed cell movement and recruitment coexist, and this process has recently been nonmechanistically defined as stem cell homing 42. Notably, the molecules that signal for cell migration can be spontaneously released in response to injury; however, in many cases such as ageing and degenerative disease, sufficient homing and healing responses are unlikely to be achieved without artificial administration of one or several key homing factors 17, 23, 24. In the next section, we will review a number of selected cell mobilization and homing factors, as well as the mechanisms for their presentation and release for enhancing *in situ* tissue regeneration.

## Chemoattractants as potent cell mobilization and homing factors

Upon inflammation or insult, MSCs respond to signals emitted by the tissue and are recruited to the inflamed or injured sites requiring repair. Some of these cues have been identified; they include but are not limited to substance P, stromal‐derived factor (SDF)‐1α, stem cell factor (SCF), granulocyte colony‐stimulating factor (G‐CSF) and monocyte chemotactic proteins (MCPs) 43. Although stem cells may be guided to promote repair based on their inherent mechanisms in response to injury, these endogenous healing processes are typically insufficient to achieve complete regeneration. Mounting evidence indicates that chemoattractants such as substance P, SDF‐1α, SCF, G‐CSF and MCP‐3 are potent stem cell‐activating factors that may be used to prolong and potentiate endogenous stem cell homing and recruitment. In particular, a growing body of preclinical investigations indicates that increasing the concentrations of some of these specific chemokines systemically and/or at sites of tissue damage, such as *via* vehicle‐aided localized delivery or biomaterial presentation, is a state‐of‐the‐art strategy to amplify wound healing and tissue regeneration 22. With respect to *in situ* tissue regeneration, the therapeutic outcome of substance P, SDF‐1α, SCF, G‐CSF and MCP‐3 will be selectively discussed in this manuscript.

### Substance P

When sensory nerves are injured, peripheral sensory neurons release substance P, which is an 11‐amino‐acid peptide and a nociceptive factor that functions as a neuromodulator and neurotransmitter and plays a role in reparative neovascularization *via* local and systemic actions 44. According to a study by Amadesi and colleagues 45, substance P‐based nociceptive signalling plays an important role in the recruitment of blood‐borne stem cells. They identified a novel regulatory mechanism triggered by limb ischaemia and involving the activation of substance P release from peripheral tissues into the circulation and the modulation (reduction) of substance P content in bone marrow 45. Substance P mediates its effects by preferentially binding and activating the tachykinin receptor NK1. Therefore, the creation of a substance P gradient between the circulation and the bone marrow facilitates the egress of neurokinin 1 (NK1)‐expressing populations from their niche into the peripheral blood; however, such reparative responses after ischaemia can be jeopardized by disruption of NK1 on bone marrow cells 45, 46. Hong *et al*. 46 showed strong evidence that substance P is a systemically acting messenger of injury that acts as a cell‐stimulating agent early in the tissue healing and repair process to induce the activation of stromal‐like CD29(+) cells from the bone marrow into the circulation in a corneal alkali burn model and to engage in tissue repair. Interestingly, a combination of the systemic injection of substance P and the local delivery of SDF‐1α from a biomatrix synergistically increased the trafficking and penetration of endogenous stem cells into the scaffold 47. In addition to systemic delivery, substance P has been widely applied in biomaterials to accelerate the endogenous recruitment of native cells for regeneration in various types of tissues 48. For *in situ* vascular regeneration, the controlled administration of substance P from the electrospun membranes of a vascular graft generated by mixing substance P‐bound poly (l‐lactide‐co‐ε‐caprolactone) (PLCL) and linear PLCL in appropriate proportions enhanced the recruitment of human BMMSCs, leading to the regeneration of abundant blood vessels in the explants 49. Similarly, in a rat knee model, self‐assembled peptide matrices coupled with substance P inhibited the progression of osteoarthritis by recruiting host MSCs to the site of insult 50. The rapid release of substance P along with the slow delivery of bone morphogenetic protein (BMP)‐2 was achieved using a heparin‐conjugated fibrin gel that enabled prompt cell recruitment during the first stage and long‐term *in situ* cell differentiation during the second stage; both features are key to ensuring effective bone regeneration 51. In a mouse model, substance P alone induced the recruitment of MSCs to the site of the ischaemic hindlimb; combined local and systemic substance P delivery resulted in a synergistic outcome, with greater cell recruitment compared with a single treatment, and effective regeneration was achieved without the injection of exogenous cells 52. Recently, substance P has been utilized in more targeted biomaterial designs. For example, a small‐diameter PLCL vascular graft with covalent binding of substance P and heparin was devised as a cell‐free strategy for *in situ* vascular regeneration; substance P was bound to recruit endogenous reparative cells, while heparin was conjugated to suppress thrombogenic responses by inducing microphages (Mφs) to polarize into the M2 phenotype 53. Aside from increasing cell migration and enhancing the egression of host MSCs into the peripheral blood, substance P increased cell proliferation and facilitated the large‐scale cultivation of cells, thereby shortening the *in vitro* propagation while sustaining the active state of MSCs 54. Furthermore, substance P exhibits the potential to rescue the weakened immunosuppressive functions of MSCs arising from long‐term culture. In this context, substance P may boost the ability of MSCs to produce TGF‐β1, thus eliminating alterations in the innate therapeutic potential of MSCs prior to their application in therapy 55. These findings suggest that substance P can play critical roles in the future *in vitro* expansion and manufacture of cellular materials.

### SDF‐1α

**Figure 3:**
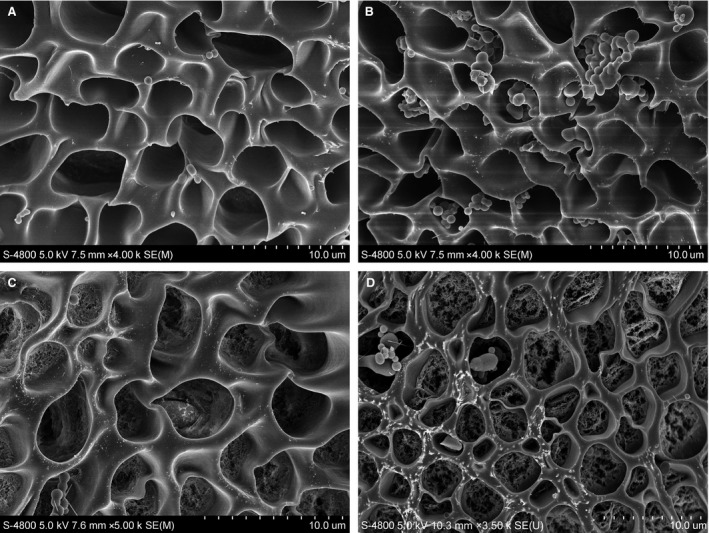
A platform featuring temperature‐controlled drug release properties due to thermo‐responsive gates grafted on their outer shell (representative SEM images of similar material devices with a tailored framework in terms of porosity and pore size as described in 62). (**A**) The macroporous pore structures of the platform; (**B**) growth factor‐loaded microparticles incorporated into the pore structures of the material; (**C**) surface engineering of pores with PNIPAAm gates; (**D**) opening of the engineered gates at temperatures above their lower critical solution temperature for drug release when PNIPAAm chains are in the shrunken state.

Under *in vivo* physiological conditions, quiescent haematopoietic stem cells (HSCs) or haematopoietic progenitor cells are maintained in bone marrow stroma through chemokine signalling between the chemotactic factor SDF‐1α, also termed chemokine (C‐X‐C motif) ligand 12 (CXCL12), and the G protein‐coupled receptor C‐X‐C chemokine receptor 4 (CXCR4) 56. In addition to confining HSCs in their proper niche, the unique SDF‐1/CXCR4 signalling has been investigated frequently due to its pivotal role in the modulation of HSC homing and the subsequent engraftment following HSC transplantation. It is now clear that the interplay between SDF‐1 and CXCR4 is an essential factor that promotes the engraftment and survival of outside infused HSCs; such pleiotropic effects render this unique signalling initiator applicable not only for the restoration of haematopoiesis but also for the design of innovative therapies to achieve regeneration of damaged tissues 57. In fact, SDF‐1/CXCR4 signalling also contributes to the homing responses of many other mesenchymal cell populations. Because a number of progenitor/stem cells migrate towards the SDF‐1 gradient, SDF‐1 has been used as a representative chemotactic factor, alone or in combination, to induce the recruitment and homing of endogenous stem cells to sites of injury within the body (reviewed in 40). In a rat model of stroke, SDF‐1α expression was increased in the ischaemic region following systemic administration of polymeric micelles incorporating SDF‐1α, thereby leading to an increase in endothelial progenitor cell (EPC) homing 58. SDF‐1 is most often administered using biomaterials as local systems rather than using systemic approaches. In this context, the incorporation of exogenous SDF‐1 into a chitosan/poly(γ‐glutamic acid) (γ‐PGA) complex, for example, can generate a high concentration gradient that drives efficient stem cell migration into the biomaterial 59. Analogously, using a knitted silk‐collagen sponge as the SDF‐1 carrier when targeting tendon regeneration can improve local endogenous SDF‐1 expression at the target site can lead to increased recruitment of fibroblast‐like cells and tendon ECM production 60. Lim *et al*. 61 engineered a multifunctional biomaterial composed of injectable hydrogels and SDF‐1α‐loaded nanoparticles for injection into cavitary brain lesions. This device offered both chemotactic cues and structural support for recruiting endogenous neural progenitor cells and enhancing neural tissue repair/regeneration 61. For the sustained and long‐term release of SDF‐1α, we designed a platform featuring thermo‐responsive drug release properties due to poly(*N*‐isopropylacrylamide) (PNIPAAm) gates grafted on its outer pore surfaces that provided a swollen‐shrunken property in response to temperature changes 62 (Fig. 3). Recently, an SDF‐1α‐loaded silk fibroin scaffold was designed that could mediate dental pulp stem cell migration and improve *de novo* pulp regeneration in pulpectomized mature teeth in a canine model 63. Likewise, SDF‐1 effectively promoted the regeneration of cartilage defects when delivered *via* a radially oriented collagen scaffold 64. When SDF‐1α was engineered with a collagen‐binding domain, intramyocardial injection of the resultant recombinant chemokine led to improved cardiac function after myocardial infarction in rats because this fusion protein and its controlled release mobilized and recruited sufficient endogenous reparative cells to the ischaemic heart 65.

For *in situ* regeneration of a specific target, SDF‐1 is commonly combined with other therapeutic agents, and cooperative effects continue to be identified; some examples include the use of SDF‐1 in combination with TGF‐β1 and BMP‐2 for site‐targeted cell trafficking and tissue‐specific differentiation 66, with BMP‐7 for tooth and periodontal regeneration 67, with simvastatin for bone regeneration 68, with an angiogenic peptide (Ac‐SDKP) for chronic myocardial infarction regeneration 69 and with a Mφ recruitment agent for enhanced wound closure in a mouse skin wound defect 70. With the aid of biomaterials and drug delivery vehicles, multiple molecules can be embedded and released in a sequential and controlled manner; hence, a synergistic effect is expected. For example, by functionalizing the scaffold with SDF‐1 *via* physical adsorption, an initial quick release of the adsorbed homing agent can lead to a rapid homing of stem cells to the implanted sites during the first few days. To modulate cell differentiation and promote bone formation, slow and long‐term release of BMP‐2 from the scaffold is required. To achieve this goal, BMP‐2 can initially be loaded into particle delivery systems (*e.g*. microspheres), and these particles are subsequently introduced into the inner structure of the scaffold; such a design ensures the release of drug for as long as several weeks. *In vivo* data have shown promise that the sequential and controlled release of these two factors from well‐designed biomaterials may be able to regenerate calvarial critical size defects in rats without cell delivery 71. Although all studies have consistently suggested that SDF‐1 can effectively attract MSCs, in the *in vivo* milieu, this chemokine tends to be present in an inactive state due to protease cleavage, normally by CD26/dipeptidylpeptidase‐4 (DPP‐4) and matrix metalloproteinase‐2 (MMP‐2). To ensure the targeting of more reparative cells to damaged tissue, Kanki and colleagues created an engineered SDF‐1 in an MMP‐2/DPP‐4‐resistant form, termed SSDF‐1(S4V), and they found that this version of SDF‐1 could not be inactivated and was highly stable. In contrast to SDF‐1, which may be cleaved by DPP‐4, direct injection of protease‐resistant SSDF‐1(S4V) into an injured rat heart recruited more reparative cells to the damaged heart, leading to a dramatic improvement in ventricular function and angiogenesis 72. A similar study by Huber and colleagues suggested that as a DPP‐4 inhibitor, parathyroid hormone (PTH) can be used to prevent DPP‐4‐induced SDF‐1 inactivation and thereby promote SDF‐1‐driven cell mobilization and homing 73. With further tuning of the release profile of SDF‐1 from biomaterial devices and their therapeutic bioactivity, this chemotactic factor may have a clinically relevant impact on tissue repair and regeneration.

### G‐CSF

G‐CSF, also termed granulocyte‐macrophage colony‐stimulating factor (GM‐CSF), was initially developed for the treatment of neutropenia after cytotoxic therapy and is approved for use in patients to prevent infection‐associated complications (*e.g*. during antineoplastic therapy) 74. However, increasing evidence has suggested that G‐CSF possesses an autocrine protective signalling mechanism for neuroprotection in response to neural injury *via* inhibiting apoptosis and inflammation. Moreover, G‐CSF may participate in neural tissue repair through stimulating neurogenesis, indicating an important non‐hematopoietic function of this biofactor 75. In fact, G‐CSF is another stem cell mobilization‐accelerating factor that stimulates the activation and egress of HSCs and BMMSCs from their niche into the bloodstream 76. After myocardial infarction, G‐CSF treatment increased the number of resident cardiac cells but reduced the capacity of BMMSCs to migrate into ischaemic tissue 77, 78. To optimize the homing capacity of BMMSCs associated with improved survival and cardiac function, G‐CSF must be combined with other agents 79. In strategies focused on *in situ* tissue regeneration, beneficial outcome was achieved *via* either local injection of G‐CSF or the incorporation of this factor into the implanted biomaterial. Local administration of G‐CSF for 14 days induced circulating EPC mobilization and recruitment to the implanted small‐diameter heparinized decellularized vascular graft, thus facilitating the generation of endothelium in the graft and the inhibition of neointimal hyperplasia 80. Similarly, single intramuscular administration of a G‐CSF‐encapsulated PEG diacrylate‐poly(ethylene imine) hydrogel scaffold extended mononuclear cell mobilization and enhanced EPC mobilization into the blood 81. In combination with G‐CSF, plerixafor (AMD3100), a CXCR4 antagonist, was evaluated in murine and human systems; the synergistic effect of AMD3100 and G‐CSF on cell mobilization led to an enhanced number of mobilized cells 82 and to significant stimulation of angiogenesis for the treatment of acute hindlimb ischaemia 83. Nonetheless, after the onset of myocardial infarction, single‐dose AMD3100 administration also increased circulating counts of EPCs and augmented their recruitment to the neovasculature, thereby improving cardiac neovascularization and functional recovery 84. Recently, the release of G‐CSF or other inflammatory cytokines from polymer matrices was shown to mediate the accumulation of dendritic cells into the material, indicating that the immune response to biomaterials can be modulated *via* controlled G‐CSF delivery 85, 86.

### SCF

SCF is a membrane‐bound soluble growth factor that is expressed on HSCs in either the primitive or the mature state. Like the c‐kit receptor CCL7 (*i.e*. chemokine (C‐C motif) ligand 7), this growth factor mediates signalling functions, including the proliferative response, cell survival and chemotactic activities. SCF synergizes with other therapeutic agents, such as G‐CSF, to augment cell mobilization and homing *in vivo* 87. SCF therapy alone can enhance CD34(+) cell yield, and when SCF therapy is combined with filgrastim, it correlates with manageable levels of toxicity for peripheral blood progenitor cell mobilization 88. Most likely *via* an autocrine pathway, the interplay between this factor and the tyrosine kinase receptor *c‐kit* plays pivotal roles in preventing vascular smooth muscle cell apoptosis, facilitating homing by circulating SCF(+) cells and developing the neointima with bone marrow‐derived c‐kit(+) progenitors 89, 90, 91. Ischaemia/reperfusion can stimulate cardiac stem cell (CSC) homing to the injured myocardium, and the accumulation of CSCs correlates with increased SCF expression 92. In this context, hyperhomocysteinemia decreases SCF expression *via* decreasing the activities of NF‐κB, ERK1/2 and p38, further inhibiting CSC recruitment into peri‐infarcted heart tissue 93. Recent work has increasingly revealed the capability of SCF to induce cell migration, angiogenesis and tissue remodelling, paving the way for its use as a potent homing agent in the regeneration of a wide variety of mesenchymal tissues (*e.g*. dental pulp) 94, 95. In combination with G‐CSF, SCF mobilized a sufficient number of BMMSCs in rat models of acute tubular necrosis and caused cell homing to the site of damage, thereby combating apoptosis and facilitating the regeneration of renal tubular epithelium following insult 79. Moreover, the precise recapitulation of native niche components, such as the covalent immobilization of SCF and SDF‐1α, has helped control the adhesion and spreading of HSCs towards their successful *in vitro* expansion 96, 97. Indeed, the modulation of SCF/c‐kit signalling controls MSC stemness and differentiation properties 98. Covalently immobilized SCF is a critical component that provides the appropriate sequence of environmental signals to selectively affect the growth of HSCs within a gelatin hydrogel in the laboratory, which is important for cell production in the treatment of blood diseases 99.

### MCP‐3

MCP‐3 belongs to the MCP subgroup of the CC chemokine family 100. By binding to different receptors, MCP‐3 activates and promotes the chemotaxis of many types of immune cells, including all types of leucocytes, dendritic cells and natural killer cells 101. MCP‐3 may induce myocardial MSC homing; MCP‐3 overexpression in freshly infarcted myocardium can recruit MSCs and improve remodelling of the cardiac collagen matrix independently of cardiac myocyte regeneration 102. Further evidence suggests that MCP‐3 may be useful for improving cardiac repair by stimulating the migration of circulating angiogenic cells and angiogenesis 103. Recent evidence suggests that the local release of MCP‐1 from instructive, bioresorbable synthetic grafts can mediate the homing of circulating cells and thereby the regeneration of small‐diameter blood vessels in rats 104.

## Regulation of stem cell migration for regeneration

The above discussion includes only a few examples of the currently investigated chemokines that demonstrate promise for inducing host stem cell recruitment. Although each signalling molecule may act on multiple cell types, stromal‐like cells in the bone marrow are more likely to be activated by substance P 45, 46, while SDF‐1α contributes largely to the homing and engraftment of HSCs in their central niche 56, 57. In contrast, G‐CSF may stimulate the release of both HSCs and BMMSCs from their niches into the bloodstream 76, 77, 78, whereas MCP‐3 induces the homing of myocardial MSCs 89, 90, 91. Specifically, SCF plays a crucial role in facilitating the homing of circulating SCF(+) cells 102, 103. Many other growth factors or biological agents, such as BMPs, TGF‐βs, insulin, fibroblast growth factors and hepatocyte growth factor, can also stimulate MSC recruitment, alone or in various combinations 66, 67, 68, 105, 106. In recent years, two reviews have specifically discussed the recruitment and homing outcome of various types of chemoattractants, including chemokines and growth factors 22, 43. In addition to those previously described chemoattractants with decisive roles in tissue‐specific reparative processes, distinct homoeostatic chemokines have also been implicated in vasculogenesis and tissue development, where they serve as guiding cues that orchestrate directional stem and progenitor cell activation and migration 107. Human MSCs migrate upon activation by CXCL8 (interleukin‐8, IL‐8) but not CCL2 (MCP‐1). Based on a 96‐well chemotaxis assay, CXCR4 and CXCR1/2 ligands (SDF‐1 and IL‐8), but not the CC chemokine receptor 2 (CCR2) ligand CCL2, have dose‐dependent chemotactic effects on human MSC recruitment 108. Using an *in vitro* model of peripheral tissue (human pancreatic islets), Sordi and colleagues showed that the released factors present in islet supernatants can stimulate chemotaxis of BMMSCs; this stimulation was further demonstrated to be largely regulated by chemokine (C‐X3‐C motif) ligand 1 (CX3CL1) and CXCL12 (SDF‐1α) 109. In cell biological assays, a distinct set of biofunctional ligands (CCL2/4/5/20, CXCL8/12 and CX3CL1) attracted BMMSCs; however, only CXCL12 (SDF‐1α) could induce cytoskeletal F‐actin polymerization 110. In addition to CCR4/7/10 and CXCR5, CXCR4 mRNA is also expressed in primary isolates of CD34(‐) progenitors and immortalized MSC lines; this SDF‐1 receptor was not detected on the surfaces of those cells 111. Apart from the SDF‐1 receptor CXCR4, MSCs isolated from bone marrow also express tyrosine kinase receptors, such as receptors for platelet‐derived growth factor and insulin‐like growth factor (IGF), as well as the RANTES and Mφ‐derived chemokine receptors CCR2/3/4 112. Based on microarray analysis, molecules such as CXCL1‐3/6/8, post‐transcriptional gene silencing 2 (PTGS2), phosphodiesterase 4B (PDE4B) and transglutaminase 2 (TGM2) are directly involved in cell migration. Other factors, such as phospholipase D1 (PLD1) and IGF‐binding protein 1 (IGFBP1), may participate in membrane and cytoskeletal reorganization. Additionally, PLD1 contributes to cell polarity, while CXCL1‐3/8 and PDE4B contribute to chemotaxis and the recruitment of cells from the bone marrow 113. Furthermore, chemotaxis assays have revealed that the chemokines CXCL11 and CXCL10 significantly attract human MSCs, while CCL16, CCL18 and CCL27 demonstrated no chemotactic effects on MSCs 114. Recently, CCL20/25 and CXCL9/16 were found to significantly enhance the transendothelial migration of MSCs across aortic endothelial cells in rats, and the transmigrated MSCs exhibited a downregulation of receptors such as CCR6, CCR9, CXCR3 and CXCR6 115.

Collectively, human BMMSCs show detectable chemotaxis towards a wide variety of chemokines, including CCL2/3/5/7/17/19–22/CCL25/CCL28, CXCL8–13/16 and CX3CL1 [43, 109, 110, 111, 112, 113, 114, 116]. Specifically, the chemokine CXCL12 (SDF‐1α) is constitutively detected in the bone marrow and probably represents the most prominent cell homing factor, attracting a wide range of stem or progenitor cells. Although the action of CXCL12 (SDF‐1α) *via* CXCR4 in the bone marrow milieu is key for retaining HSCs in a quiescent state and maintaining a number of distinct cell populations, the molecular events involved in cellular chemotaxis and migration remain largely unknown 117. Currently, 30 differentially expressed genes (6 repressed and 24 induced) have been detected by microarray analysis, and 11 of these differentially expressed genes are involved in the molecular pathways of cytokine‐cytokine receptor interactions and cellular movement 118. Notably, CXCL12 (SDF‐1α) also signals through the receptor CXCR7; however, no evidence supports the involvement of CXCR7 in signalling pathways that mediate cell migration 119.

Regulation of the selective mobilization of subsets of progenitors from the bone marrow, such as endothelial and stromal progenitors, depends on the cytokine microenvironment, which modulates cell retention and proliferation. By disrupting the CXCR4/SDF‐1α axis, for example, G‐CSF stimulates HPC release into the bloodstream 76. Following pre‐treatment of mice with vascular endothelial growth factor (VEGF), the CXCR4/SDF‐1α retention axis was not disrupted, and HPCs were not mobilized, but the entry of these cells into the cell cycle was stimulated *via* VEGF receptor 1 120. In contrast, enhanced EPC mobilization *via* VEGF receptor 2 occurs in response to CXCR4 antagonism following VEGF pre‐treatment. Furthermore, in VEGF‐pre‐treated mice, administration of a CXCR4 antagonist produced detectable stromal progenitor cell mobilization, whereas G‐CSF administration did not 120, 121. The regulatory mechanisms underlying cell activation and recruitment from bone marrow can be further exploited to develop efficacious therapeutic paradigms that harness discrete populations of stem/progenitor cells for tissue regeneration.

**Figure 4:**
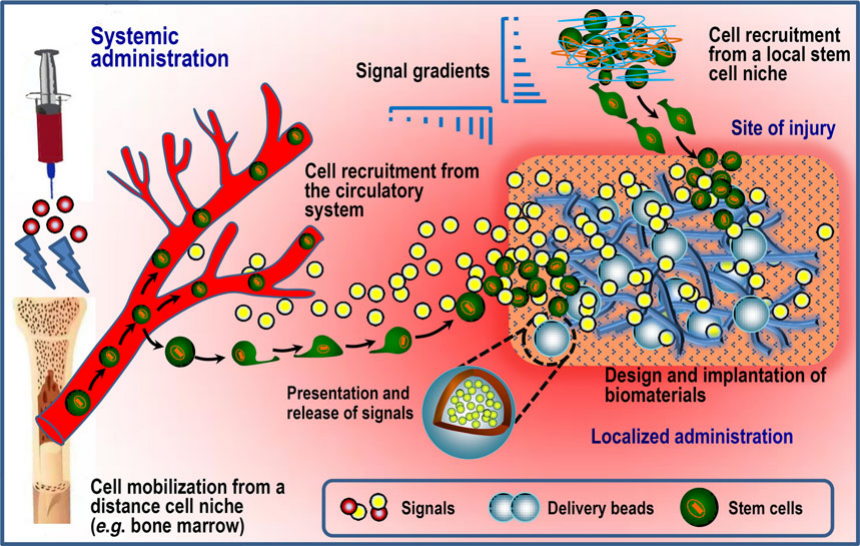
Schematic representation of strategies employed for the systemic administration of chemoattractants and chemical signals to mobilize and recruit stem cells from the circulatory system and/or the localized presentation and release of homing factors at the site of injury. Localized administrated signals can coax stem cell migration from neighbouring healthy tissue based on signalling gradients that are commonly established by the implantation of a well‐designed biomaterial platform. Endogenous cells recruited from either the circulatory system or a local cell niche can participate in tissue regeneration at the injured site.

In the last decade, much of our current knowledge on stem cell homing and accommodation has been limited to how soluble biofactors, such as chemokines and cytokines, influence resident cells. Strategies for the administration of chemoattractants and chemical signals systemically and/or at the desired destination are critically important for enhancing the endogenous regeneration process because the presentation and release of signalling factors make the site of injury and/or inflammation more attractive to stem cells and hence can dictate stem cell homing 40, 122, 123 (Fig. 4). Guan and colleagues developed a peptidomimetic ligand named LLP2A that demonstrates high specificity and affinity for activated α4β1 integrin 124. Integrins are critical for cell movement and play essential roles in directing HSCs to bone 125. Therefore, the injection of the ligand LLP2A may drive MSCs to the surface of bone. When this ligand was further conjugated to alendronate to engineer the hybrid compound LLP2A‐Ale and tested in animals, the rate of bone formation in both xenotransplantation studies and immunocompetent mice was significantly increased *via* a single intravenous administration of LLP2A‐Ale 124. Because alendronate is a bisphosphonate with high affinity for bone, it functions as the bone‐tracking constituent that can recruit LLP2A to bone 126, 127. Indeed, the promotion of cell recruitment and regeneration can also be achieved *via* the co‐delivery of silicon ions and growth factors, which may exert a synergistic effect on endogenous stem/progenitor cells 128.

**Figure 5:**
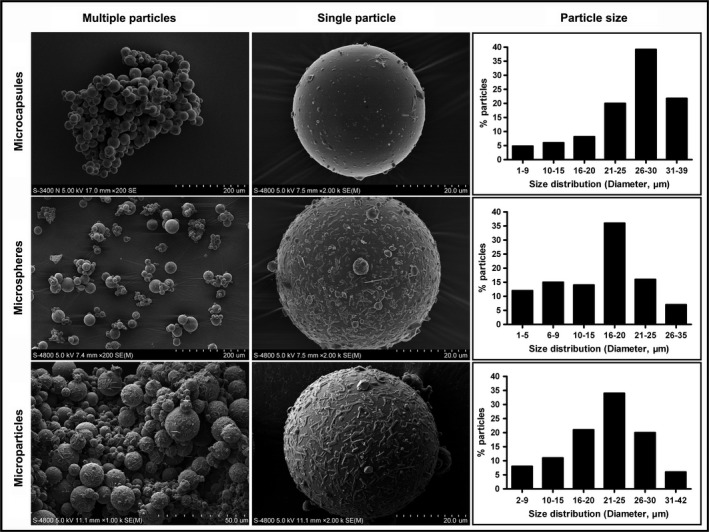
Microparticulate delivery systems for the controlled presentation and release of various bioactive factors for scaffold development and/or regenerative therapy applications (representative SEM images of similar microparticulates fabricated in our laboratory with tailored particle sizes as described in 19, 62, 129, 130).

Recently, extraordinary accomplishments have been achieved in the development of biomaterial vehicles that are capable of incorporating and releasing selected chemokines for therapeutic tissue regeneration based on cell recruitment and homing 17, 22. However, scientific and technological challenges in material design and drug delivery must be overcome to obtain an accurate mimic of the natural wound healing cascade. Although a wide range of developed drug delivery systems may also be used for the presentation and release of homing factors (*e.g*. see Fig. 5) 62, 129, 130, new insights into the effects of the doses of chemokines and their concentration gradients on cell mobilization and trafficking will enable the precise development of future endogenous regenerative therapies 131. Moreover, the complexity of the *in vivo* milieu underscores the importance of considering not only the correlation among cargo release kinetics, optimal time‐points and cell recruitment efficacy but also other environmental factors such as ECM stiffness, architecture and composition, which can jointly impact stem cell fates. Indeed, new findings continue to generate concern regarding how biomaterial cues may control cell geometry and how a diverse array of biophysical factors may be transmitted into the cell 132. Thus, regulating cell activity requires the reestablishment of an *in vivo* environment with a suitable hierarchical structure and topography at the nanoscale level and with proper mechanical properties 133.

## Increasing the surface sensitivity of stem cells to homing factors

As an alternative to delivering chemical signals, ions or other chemokines through a biomaterial platform, augmented cell homing can be achieved by making the surfaces of the targeted cells more responsive to specific homing inducers, typically *via* chemical modification and/or genetic engineering 134, 135, 136. The development of tools for enhancing cell retention is feasible for increasing targeting efficiencies and has become the research focus of many current endeavours in cell‐based therapy 137, 138. Although cell modification cannot be incorporated into *in situ* tissue‐engineering strategies without transplanting *ex vivo*‐expanded stem cells, this strategy offers an important tool for studying or regulating *in vivo* cell homing processes and would be useful for the future design of endogenous regenerative approaches 139. On the other hand, it is clear that homing of the patient's own cells may be restricted by ageing, inflammation or disease. In the ageing population, for example, the number of resident reparative cells is intrinsically insufficient for mobilization, and those cells may intrinsically have poor migration and regenerative potentials for tissue regeneration. In such cases, the delivery of outside expanded cells would be necessary to achieve a therapeutically regenerative solution 74. Interestingly, there is evidence that cell transplantation can act as an initiator to trigger endogenous regenerative process, typically *via* the secretion of growth factors or various other cytokines that enhance migration of endogenous stem cells to the lesion site and improve their function for integration, angiogenesis and neovascularization 140, 141, 142. In this respect, cell transplantation would not be excluded from the field of *in situ* tissue regeneration.

Systemic infusion is a typical and convenient strategy that maximizes practical aspects of repeated doses and minimizes the invasiveness of cellular therapy; however, this delivery mode cannot guarantee engraftment efficiency 137, 138. For clinical applications of adoptive cellular therapy to be successful, the ability to deliver a sufficient number of viable cells to a predetermined anatomical compartment with a high efficiency of cell engraftment or the ability to drive the recruitment of blood‐borne cells to sites where they are needed is critical and presents a substantial challenge 143. In local implantation models, the expression of specific homing factors has also shown promise for enhancing the homing of culture‐expanded osteogenic cells into bone fracture sites 144. In some cases, cells are genetically modified *via* the introduction of specific gene sequences prior to transplantation; once these cells live in an *in vivo* milieu, they may express factors that induce recruitment, subsequently promoting the homing of endogenous stem cells 40. For example, the use of an adenovirus to overexpress SDF‐1 in stem cells increased the effectiveness of native cell trafficking to the bone defect and hence led to enhanced bone regeneration at the site of fracture by promoting osteogenic differentiation and bone production 145. Analogously, SDF‐1 has a stimulatory effect on stem cell recruitment to the bone defect when using adipose tissue grafts that were adenovirally activated to express SDF‐1α and/or BMP‐2 146. Further, gene‐activated scaffolds delivering both VEGF and BMP‐2 produced much greater vascularization and bone regeneration than did either a single delivery or a blank control system 147. Although cells overexpressing other factors have been used as therapeutics in a number of similar studies, insufficient evidence supports enhanced tissue regeneration as an outcome of increased recruitment of host cells in response to their transplantation 69, 148, 149. Indeed, genetically engineered cells that exhibit prolonged expression of therapeutic agents themselves may have an enhanced regenerative potential at the site of action 150.

Although modification of cellular binding sites may be a viable approach to facilitating cell homing to a tissue of interest, current strategies, such as gene transfection, are practically complex and have potential safety concerns 137. In addition, these approaches do not offer a universal solution; *that is*, they are either not adaptable to different type of cells or unable to accommodate a vast array of ligand molecules. Immense effort is currently focused on making genetic modification simple and safe. Using lipid vesicles, Sarkar and colleagues reported a simple method to transiently modify cell surfaces, resulting in the ability to efficiently immobilize molecular ligands on cell surfaces for targeting to the inflamed site following systemic administration 134. Likewise, preconditioning stem cells with Ro‐31‐8425, an identified hit from a small‐molecule screen, increases firm cell adhesion to an ICAM‐1‐coated substrate *in vitro*. Further experiments demonstrated that such chemical modification enabled the systemic infusion and targeted recruitment of MSCs to an inflamed region *in vivo* in a CD11a‐dependent (and other ICAM‐1‐binding domain‐dependent) manner 151. Both studies imply that surface modifications of MSCs may help the cells find their way to a tissue of interest or to the bone marrow and potentially yield high‐efficiency targeting of other cell types to specific tissue areas *via* the bloodstream 135.

For stem cells to successfully home to sites of injury, a sequence of coordinated and regulatory interplays between a cell and its microenvironment provides signs that the signal is reaching the cell along its journey. Chemokines are the most important factors that regulate cell migration *in vitro* and *in vivo*, and MSCs move in response to a CXCL12 (SDF‐1α) gradient 22, 117. However, the *in vitro* chemotactic response of human MSCs to thymus‐expressed chemokine (TECK), or CCL25, is more than 10‐fold greater than that to CXCL12 (SDF‐1α), indicating that TECK is a more potent *in vitro* chemoattractant for MSCs 113. MSCs without preconditioning show very low CXCR4 surface expression; hence, the *in vitro* SDF‐1‐directed migration potential for human MSCs is quite low 111. However, the chemokine receptor expression and chemotaxis of MSCs can be increased by shear stress or by hypoxic/proinflammatory preconditioning 112. Hypoxic preconditioning of BMMSCs before transplantation increases the expression of CXCR4/7; facilitates *in vitro* cell adhesion, migration and survival; and ameliorates the cells’ capacity to survive and engraft at the site of interest 152. Further results indicate that hypoxia inducible factor (HIF)‐1α plays very important roles in hypoxia‐induced cell activation and movement, most likely acting *via* its downstream genes SDF‐1α and VEGF 153. Additionally, short‐term stimulation of BMMSCs with combinations of various cytokines demonstrated up‐regulated cell surface and intracellular CXCR4 expression, leading to improved cell trafficking potential *in vitro* and *in vivo* 154. Although numerous investigations have revealed that either a hypoxic or an inflammatory stimulus may increase the capacity for cell migration, dual stimuli combined with hypoxia and inflammation do not necessarily lead to a synergistic effect 155, 156. Further strategies to increase and control cell migration *in vivo* and to control this process may bring us closer to reaching the original goals of stem cell therapy.

## Challenges and opportunities

The rising incidence of tissue insult caused by trauma, infection and/or destructive disease has led to an increasing demand for new, safe, effective and practical therapies for clinical use; this situation is exacerbated by an increasingly ageing population. In this context, the last two decades have witnessed remarkable laboratory‐based and preclinical success in the development of tissue‐engineering therapies 157, 158, 159. Unfortunately, very few of these novel therapeutics are being used in clinical applications 160. For clinical translation and population‐wide applications, constructs engineered in the laboratory based on stem cells in combination with signalling factors and/or material scaffolds will probably be deemed unrealistic because of the time, cost and indeed numerous regulatory issues 122, 161. *In situ* tissue regenerative procedures offer great potential for broader clinical application and, more importantly, would only rely on off‐the‐shelf scaffolds without the need for *ex vivo*‐manipulated stem cells, thus reducing the time and cost in comparison with the classical tissue‐engineering approach 24, 159. With the aid of signalling molecules, such biomaterial devices should mimic the native nanoenvironment of regenerating tissues as closely as possible and thereby unlock the innate regenerative capacities of the body for therapeutic regeneration applications 159, 161, 162.

Functionally, issues remaining to be addressed include the chemotaxis of reparative stem cells following material transplantation, the amplification of these cells (*via* proliferation) as a transient pool, and the local functions of these cells within the materials, particularly considering secretion, concerted differentiation and remodelling 22. Increasing the migratory capacity of reparative cells, the sensitivity of the target sites and hence the efficiency of stem cell homing is important for accelerating tissue repair and regeneration. Unfortunately, we still do not know which cell type is most likely to be recruited to a site of injury or how different cell types collaborate during cell homing and regeneration. Therefore, further investigation into the mechanisms underlying both stem cell homing and cell‐mediated regeneration may not only facilitate the development of effective yet simple endogenous regenerative therapies but also eventually expedite progress in many related fields, such as biomaterials science and *in situ* tissue engineering 74. Intensive effort has been and continues to be directed towards this field, but success is not a simple proposition. In terms of chemokine presentation, we need to control the interaction between the incorporated protein and the material in order to slow their diffusive release, considering the protein itself, the vehicle for delivery and the microenvironment targeted for therapy 162, 163. With respect to cell movement, numerous static and dynamic (transendothelial) *in vitro* migration models have been used to study cell chemotaxis; however, a discrepancy between primary and *ex vivo‐*expanded MSCs in terms of mobilization or homing has been identified based on the observation that the cell phenotype undergoes dramatic changes during the *in vitro* expansion phase 164. Currently, no innovative culture system can better characterize different cell types in their native and *in vitro* environments, and we still lack a reliable approach for *in vivo* monitoring of cell trafficking to an injury site. Based on *in vivo* microscopy, labelled cells can now be tracked in an animal after transplantation. Intravital microscopy can visualize labelled cells in a given tissue or organ after surgical dissection, thus revealing the *in vivo* chemokine‐directed interactions between MSCs and the endothelium and the subsequent cell migration within the ECM 22, 165. However, we still cannot identify native MSCs and track their chemotaxis *in situ*, and the actual roles of endogenously recruited cell populations in tissue regeneration remain unclear 166. Advancements in *in vitro* and *in vivo* model investigations will be rewarded with increased potential across the arena of stem cell biology and therapy 137, 167.

As the implantation of outside devices into the *in vivo* milieu often produces inflammatory states, insights into the impacts of a variety of environmental stimuli generated by implants on Mφ phenotype and function may offer new information for future materials design 168, 169, 170, 171. Because the cellular response to chemokines is affected by the pathophysiological state and the status of the tissue insult, another exciting and emerging area related to the endogenous regeneration paradigm is immuno‐engineering, in which material devices can be utilized to modulate immune responses (*i.e*. the balance between immunosuppressive and immunostimulatory responses), to therapeutically stimulate receptor complexes and cells, or as vaccines for the delivery of multiple immunomodulatory molecules 172. Directed stem cell homing thus provides a new avenue for research into regeneration, and the combination of immuno‐engineering and *in situ* tissue engineering will continue to provide new and more effective and treatments to address the problems currently encountered in the clinic 161, 169.

## Conclusions

The local use of signalling molecules at the area of injury to drive stem cell homing *in vivo* is a new therapeutic strategy that holds great potential for achieving functional tissue regeneration without the need for the delivery of *ex vivo*‐expanded cells. To exploit homing mechanisms therapeutically, we must identify the complex interactions and pathways underlying the cascades of cellular events involved in wound healing and regeneration. Additionally, suitable animal models for tracking and evaluating endogenous reparative cells *in vivo* must be established to reveal the chemokine signalling and activity underlying directed cell recruitment. For this strategy to be successful, further investigations are required to ensure that the recruited cells propagate and divide into appropriate cell types for regeneration. In conclusion, the regulation of stem cell homing as a new paradigm for regeneration is still in its infancy. Further optimization of current material platforms that deliver potent signals for stem cell mobilization and homing and a better understanding of the priming or upstream signals involved in functional *in situ* tissue regeneration are necessary before an effective therapeutic outcome can be translated into the clinic for patient health care.

## Authors’ contributions

All authors participated in the drafting, writing and revision of the manuscript and gave final approval for publication of the work.

## Ethics approval and consent to participate

Not applicable.

## Conflict of interests

The authors confirm that there are no conflict of interests.
